# Development of Prediction Model for Intensive Care Unit Admission Based on Heart Rate Variability: A Case–Control Matched Analysis

**DOI:** 10.3390/diagnostics14080816

**Published:** 2024-04-14

**Authors:** Dong Hyun Choi, Hyunju Lee, Hyunjin Joo, Hyoun-Joong Kong, Seung Bok Lee, Sungwan Kim, Sang Do Shin, Ki Hong Kim

**Affiliations:** 1 Department of Biomedical Engineering, Seoul National University College of Medicine, Seoul 03080, Republic of Korea; donghyun369@naver.com (D.H.C.); sungwan@snu.ac.kr (S.K.); 2 Laboratory of Emergency Medical Services, Seoul National University Hospital Biomedical Research Institute, Seoul 03080, Republic of Korea; llhj2021@gmail.com (H.L.); sdshin@snu.ac.kr (S.D.S.); 3 Innovative Medical Technology Research Institute, Seoul National University Hospital, Seoul 03080, Republic of Korea; a74741234@gmail.com (H.J.); gongcop7@snu.ac.kr (H.-J.K.); 4 Department of Transdisciplinary Medicine, Innovative Medical Technology Research Institute, Seoul National University Hospital, Seoul 03080, Republic of Korea; 5 Department of Medicine, Seoul National University College of Medicine, Seoul 03080, Republic of Korea; 6 Medical Big Data Research Center, Seoul National University College of Medicine, Seoul 03080, Republic of Korea; hisblee@snu.ac.kr; 7 Institute of Bioengineering, Seoul National University, Seoul 08826, Republic of Korea; 8 Department of Emergency Medicine, Seoul National University Hospital, Seoul 03080, Republic of Korea

**Keywords:** intensive care units, forecasting, heart rate variability, emergency service, case–control studies

## Abstract

This study aimed to develop a predictive model for intensive care unit (ICU) admission by using heart rate variability (HRV) data. This retrospective case–control study used two datasets (emergency department [ED] patients admitted to the ICU, and patients in the operating room without ICU admission) from a single academic tertiary hospital. HRV metrics were measured every 5 min using R-peak-to-R-peak (R-R) intervals. We developed a generalized linear mixed model to predict ICU admission and assessed the area under the receiver operating characteristic curve (AUC). Odds ratios (ORs) with 95% confidence intervals (CIs) were calculated from the coefficients. We analyzed 610 (ICU: 122; non-ICU: 488) patients, and the factors influencing the odds of ICU admission included a history of diabetes mellitus (OR [95% CI]: 3.33 [1.71–6.48]); a higher heart rate (OR [95% CI]: 3.40 [2.97–3.90] per 10-unit increase); a higher root mean square of successive R-R interval differences (RMSSD; OR [95% CI]: 1.36 [1.22–1.51] per 10-unit increase); and a lower standard deviation of R-R intervals (SDRR; OR [95% CI], 0.68 [0.60–0.78] per 10-unit increase). The final model achieved an AUC of 0.947 (95% CI: 0.906–0.987). The developed model effectively predicted ICU admission among a mixed population from the ED and operating room.

## 1. Introduction

The number of emergency department (ED) visits has surged over the past several decades, with approximately 150 million annual visits recorded in the United States, and 10 million visits recorded in Korea [1,2,3]. This increase has given rise to challenges such as overcrowding in EDs and resource shortages [4]. Furthermore, ED crowding is associated with the delayed detection of patients deteriorating into critical conditions while awaiting treatment [5]. Moreover, the availability of ED beds for patient monitoring is constrained, and a limited number of health care providers are available to check the status of all patients. Hence, it is crucial to develop accessible methods for identifying critically ill patients who are not under rigorous monitoring.

Heart rate variability (HRV) quantifies the fluctuation in time intervals between successive heartbeats and can be assessed using various methods. Time domain metrics include the standard deviation of R-R intervals (SDRR) and the root mean square of successive R-R interval differences (RMSSD). The frequency domain metrics include low-frequency (LF) power, high-frequency (HF) power, and LF/HF ratio. While HRV can be assessed over ultra-short-term (less than 5 min), short-term (approximately 5 min), and long-term (approximately 24 h) durations, short-term measurement is most commonly employed due to its convenience in data acquisition and its ability to capture slow fluctuations in heart rate [6].

HRV is recognized for its representation of cardiovascular function and autonomic balance. It has been identified to be associated with various severe illnesses, which are accompanied by a decline in cardiovascular function and a disruption in autonomic balance [7,8,9,10]. One study demonstrated that a low SDRR predicted mortality in sepsis patients in the intensive care unit (ICU) [9]. Another study demonstrated that a decrease in the LF and an increase in the HF signal were associated with the severity of sepsis in patients in the ED [7]. In another study, multiple HRV metrics were used to predict in-hospital cardiac arrest [10]. As demonstrated in previous research, HRV serves as a pivotal marker of physiologic compensation in critically ill patients.

The accurate identification of critically ill patients in the ED who require ICU admission is crucial for ensuring early treatment and adequate preparation of ICU resources. Therefore, the aim of this study was to develop and validate a model using heart rate (HR) and HRV data to predict ICU admission. By utilizing a concise set of variables that can be easily obtained through electrocardiography (ECG) or photoplethysmography (PPG) sensors, we developed a model applicable to patients not undergoing comprehensive vital sign monitoring.

## 2. Materials and Methods

### 2.1. Ethical Statement

This study was conducted according to the guidelines of the Declaration of Helsinki and approved by the Institutional Review Board of Seoul National University Hospital (IRB number: 2307-147-1452; date of approval: 28 July 2023). Patient consent was waived due to its retrospective design and the anonymization of patient data.

### 2.2. Study Design and Setting

This retrospective case–control study utilized two datasets derived from a single urban tertiary hospital in Seoul, South Korea. The ED-VitalDB dataset encompasses adult (18 years or older) ED patients triaged to the highest acuity (level 1) and subsequently assigned to the resuscitation room. The study hospital uses the Korean Triage and Acuity Scale (KTAS), which is a 5-level ED triage scale developed based on the Canadian Triage and Acuity Scale [11]. Continuous PPG and ECG monitoring were applied to most of the patients who entered the resuscitation room. Invasive monitoring (including arterial blood pressure) was applied and treatment decisions were made at the discretion of the attending ED physician. Vital sign data were recorded using VitalRecorder (Ver. 1.11.12.0, accessed on 20 December 2021), which is a free software designed for recording biosignal waveforms and vital signs [12]. Trained reviewers retrieved patient demographic, ED evaluation, diagnosis, management, and disposition data by reviewing hospital medical records. If vital sign data corresponding to a patient were identified, the patient’s data were incorporated into the ED-VitalDB dataset after anonymization.

The OR-VitalDB dataset (available at https://vitaldb.net/dataset/ accessed on 20 December 2021) is an open dataset comprising patient data from operating rooms of the study institution collected from August 2016 to June 2017 [13]. This dataset encompasses vital sign data recorded during surgery, patient demographics, surgical details, and outcomes.

### 2.3. Study Population

Among the patients in the ED-VitalDB dataset, individuals who presented to the ED from April 2018 to December 2021, who were triaged to level 1, and who were admitted to the ICU were designated case patients. Additionally, patients who died in the ED were categorized as ICU-admitted patients. We excluded individuals with less than 5 min of ECG waveform data, those in a cardiac arrest state, those transferred to another hospital, and those aged 75 years or older.

Individuals in the OR-VitalDB dataset without critical conditions served as the control group. Patients with less than 5 min of ECG waveform data, those who underwent emergency surgery, individuals admitted to the ICU, those who died in hospital, and those aged 75 years or older were excluded from the analysis. The rationale for selecting patients from the OR-VitalDB dataset as the control group was that noncritical patients with stable vital signs are usually not subject to continuous vital sign monitoring in the ED.

Case–control matching was performed using a 1:4 ratio based on age (<20, 20–24, 25–29, …, 70–74 years) and sex. The decision to exclude patients 75 years or older was made due to an insufficient number of individuals within this age range in the OR-VitalDB dataset, thus preventing a complete match.

### 2.4. Variables and Measurements

For each patient, we calculated the SDRR, RMSSD, normalized LF power, normalized HF power, LF/HF ratio, and HR at 5 min intervals for up to two hours following ED arrival (for case patients) or at the start of the operation (for control patients). HRV was calculated through the following steps. Initially, the Lead II ECG signal was sampled at 125 Hz, and intervals were removed if no meaningful ECG signal was observed upon visual inspection. Subsequently, the ECG signal was split into 5 min intervals and passed through a 0.5 Hz fifth-order Butterworth highpass filter to eliminate noise. Afterwards, the R peaks were identified, and the RR intervals were measured. Finally, the SDRR, RMSSD, normalized LF power, normalized HF power, and LF/HF ratio were computed from the RR intervals by using the NeuroKit2 Library [14]. The normalized LF and HF power were determined by calculating the percentage of LF and HF power in relation to the total power, respectively. The LF/HF ratio was calculated by dividing the LF power by the HF power.

Information including age, sex, comorbidities, cause of ICU admission, intubation status, time of ED arrival, and time of intubation in the case group was acquired through medical record review performed by trained medical record reviewers. Demographic information for the control group patients was obtained from the OR-VitalDB dataset.

### 2.5. Model Development

The case–control matched dataset was randomly divided into a derivation set (75%) and a validation set (25%). Therefore, patients did not overlap between the two sets. The derivation set was exclusively utilized for constructing the model, whereas the validation set was reserved solely for the purpose of validating the model’s performance.

Given the repeated 5 min interval HR and HRV measurements for each patient, we constructed a generalized linear mixed model (GLMM) using all of the available repeated measurements. The following variables were considered fixed effects for the model: age, sex, hypertension history, diabetes mellitus history, HR, SDRR, RMSSD, normalized LF power, normalized HF power, and LF/HF ratio. Backward elimination was employed to eliminate nonsignificant variables. Consequently, we developed a GLMM (Model 1) that included diabetes mellitus, HR, SDRR, and RMSSD as fixed effects, with patients treated as a random effect.

A previous study demonstrated improved predictive performance for sepsis severity with HRV metrics adjusted by reference values of the same age and sex [7]. By using a similar approach, we computed adjusted SDRR and RMSSD values by subtracting the reference values of the same age and sex from a patient’s SDRR and RMSSD values. The reference values were obtained from a previous study that analyzed 5 min HRV measurements of 8 million individuals (Appendix A) [15]. As a result, another GLMM (Model 2) that included diabetes mellitus incidence, HR, adjusted SDRR, and adjusted RMSSD as fixed effects was constructed, with patient treatment as a random effect.

### 2.6. Outcomes

The main outcome of this study was the area under the receiver operating characteristic curve (AUC) of the models for predicting ICU admission. Secondary outcomes included the sensitivity and specificity of the models. Predicting ICU admission is equivalent to predicting case patients, as all case patients were admitted to the ICU or faced ED mortality, whereas none of the control patients were admitted to the ICU or faced in-hospital mortality.

### 2.7. Statistical Analysis

Categorical variables are presented as numbers and proportions, and comparisons were conducted by using the chi-square test. Continuous variables are expressed as medians and interquartile ranges (IQRs), and comparisons were made by using the Wilcoxon rank-sum test. The mean values of the repeatedly measured HR and HRV metrics for each patient were compared between groups. Odds ratios (ORs) with 95% confidence intervals (CIs) were analyzed by using the coefficients of the fixed effects of the GLMM. The variance inflation factor (VIF) was assessed to evaluate multicollinearity among the included variables.

We calculated the AUC, sensitivity, specificity, and 95% CIs by using the following three approaches with data from the validation set: the first data point for each patient, the last data point for each patient, and a single randomly sampled data point at any time for each patient. Sensitivity and specificity were determined by maximizing Youden’s index. Model calibration was assessed by using calibration plots.

We assessed the applicability of the developed models in scenarios where HR and HRV metrics were measured by using a PPG sensor. By adopting the approach of a previous study, we estimated the beat-to-beat intervals from 5 min PPG signals. The subsequent steps paralleled those used for obtaining HRV metrics from an ECG sensor.

We conducted a sensitivity analysis focusing solely on patients in the derivation set who were intubated and sedated. This was to determine whether the association between HRV metrics and ICU admission persisted regardless of intubation and sedation. For the case group, only patients who were intubated (with sedation) were selected, and HR and HRV data collected post-intubation were analyzed. In the control group, we included only the data from patients who were under general anesthesia. GLMMs using the same variables as Model 1 and Model 2 were fitted, and the ORs with 95% CIs of the fixed effects were analyzed.

A two-sided *p* value less than 0.05 was considered to indicate statistical significance. Statistical analyses were performed by using Python (Python Software Foundation, Wilmington, DE, USA) version 3.9.16, NeuroKit2 version 0.2.7, and SAS version 9.4 (SAS Institute, Inc., Cary, NC, USA).

## 3. Results

**Figure 1:**
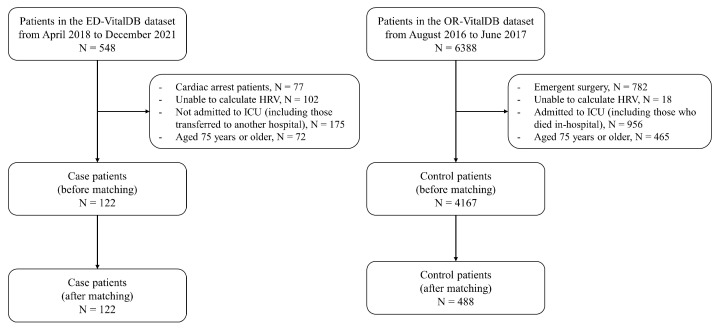
Study flowchart. Abbreviations: HRV, heart rate variability; ICU, intensive care unit.

A total of 122 patients from the case group and 4167 patients from the control group met the inclusion criteria. Among these, 122 patients from the case group and 488 patients from the control group were matched and included in the analysis, respectively (Figure 1). The case group exhibited a greater mean HR (median [IQR]: 105.4 [91.5–116.7] vs. 69.5 [63.3–77.1]) and lower mean SDRR (median [IQR]: 41.6 [24.3–61.8] vs. 66.2 [45.3–89.2]) and RMSSD (median [IQR]: 51.0 [25.6–81.0] vs. 60.3 [35.1–90.2]) than the control group (Table 1).

Among the 122 patients in the case group, 37 patients were admitted due to sepsis, 15 due to respiratory causes, 27 due to major bleeding, 22 due to major trauma, and 21 due to other causes. Among the 488 patients in the control group, 458 (93.9%) underwent surgery under general anesthesia, whereas 30 (6.1%) underwent surgery under spinal anesthesia. Demographics and vital sign measurements of patients in the derivation (*n* = 457) and validation (*n* = 153) sets showed no significant differences (Appendix B).

By analyzing the coefficients of the GLMM, we observed that a history of diabetes mellitus (OR [95% CI]: 3.33 [1.71–6.48]), a higher HR (OR [95% CI]: 3.40 [2.97–3.90] for every 10 increase), and a higher RMSSD (OR [95% CI]: 1.36 [1.22–1.51] for every 10 increase) increased the odds of ICU admission. In contrast, a higher SDRR decreased the odds of ICU admission (OR [95% CI]: 0.68 [0.60–0.78] for every 10 increase). Although the SDRR and RMSSD demonstrated a positive correlation, the VIFs for all of the fixed-effect variables remained below 10, thus suggesting the absence of significant multicollinearity (Appendix C). The ORs remained similar when age- and sex-adjusted values were used for the SDRR and RMSSD (Table 2).

**Figure 2:**
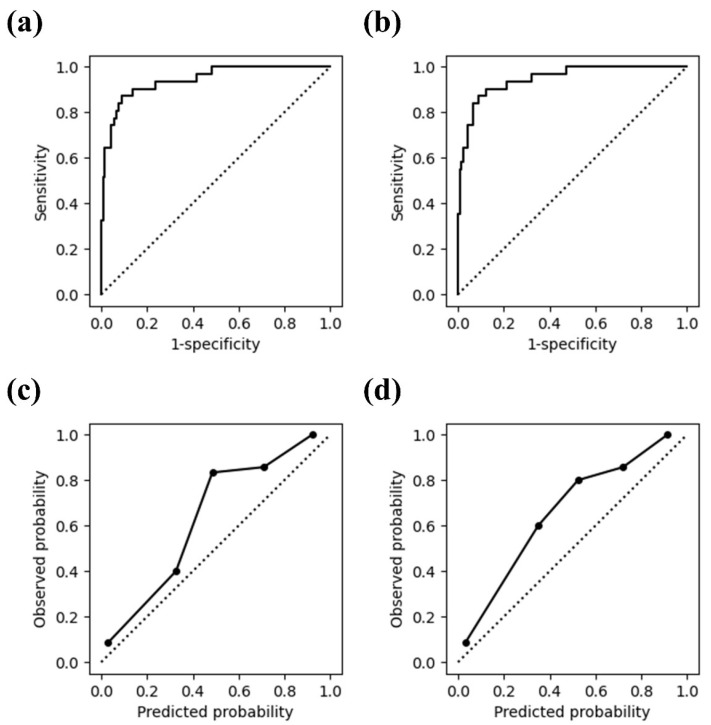
Receiver operating characteristic curves and calibration plots for (**a**,**c**) Model 1 and (**b**,**d**) Model 2.

When evaluated by using randomly sampled data points from each patient in the validation set, the AUC for predicting ICU admission was 0.942 (95% CI: 0.897–0.987) for Model 1 and 0.947 (95% CI: 0.906–0.987) for Model 2 (Figure 2a,b). The sensitivity and specificity of both models were 0.871 (95% CI: 0.753–0.989) and 0.910 (95% CI: 0.859–0.961), respectively (Table 3). The models tended to slightly underestimate the probability within the predicted probability range of 0.4 to 0.8 (Figure 2c,d).

With respect to the validation set of 153 patients, we successfully acquired PPG-derived HRV measurements for 133 patients. The discriminative performance of the models was consistent when these PPG-derived metrics were used instead of ECG-derived HRV data. When evaluated with randomly sampled data points from each patient, the AUC for predicting ICU admission was 0.928 (95% CI: 0.855–1.000) for Model 1 and 0.926 (95% CI: 0.853–0.998) for Model 2 (Table 4).

In the sensitivity analysis focusing on patients who were intubated and sedated in the derivation set, 494 data points from 29 patients in the case group and 6242 data points from 346 patients in the control group were analyzed (Appendix D). Although statistically not significant, a history of diabetes mellitus, higher HR, higher RMSSD, and lower SDRR were associated with increased odds of ICU admission, demonstrating a trend consistent with the main analysis (Appendix E).

## 4. Discussion

In this retrospective case–control matched analysis, we developed GLMMs that utilized patient demographics, HR, and HRV data to predict ICU admission. We found that a history of diabetes mellitus, a higher HR and RMSSD, and a lower SDRR were associated with increased odds of ICU admission. The models accurately predicted ICU admission, achieving an AUC of 0.88–0.95. Although these models were initially developed by using HRV metrics derived from ECG signals, they also performed accurately with HRV metrics obtained from PPG signals. Given that the models require only a simple set of demographic information and vital signs acquired from a single PPG or ECG sensor, they can be easily adopted in various settings.

The SDRR is known to reflect both sympathetic and parasympathetic nervous system activities, with lower values linked to poorer health outcomes. The RMSSD, which is strongly correlated with HF power, is predominantly influenced by the parasympathetic nervous system [6]. Previous research has indicated that sympathovagal balance is disrupted in severely ill patients. In such patients, decreased sympathetic activity impairs the body’s ability to respond adequately to stressors, such as maintaining normal blood pressure. Conversely, an increase in the relative strength of the parasympathetic nervous system is observed, which is associated with the severity of the illness [7,16]. The findings of our study, which showed that a higher RMSSD and lower SDRR were correlated with increased odds of ICU admission, align with these previous studies.

Given that our study employed a case–control design with patients matched by age and sex, these factors were not significant predictors according to Model 1. Nevertheless, age and sex are widely recognized as influencing ICU admission risk and are known to correlate with HRV values [15,17]. Model 2, which adjusts for age and sex in the SDRR and RMSSD calculations, could address this limitation of Model 1. Although Model 2 did not show significantly enhanced performance in our validation set compared to Model 1, it is anticipated to be more robust in populations with different age and sex distributions.

Our study included a sensitivity analysis to account for the impact of sedation and intubation on HRV parameters, as these procedures can alter autonomic balance and potentially skew the association with ICU admission risk [18,19]. By isolating the subset of patients who received both interventions, we aimed to ensure that our findings on the predictive value of HRV for ICU admission were not confounded by these factors. Although we did not observe statistically significant results due to a reduced sample size, the trend of association between HRV parameters and ICU admission was maintained. This result strengthens the utility of HRV as a biomarker for assessing ICU admission risk, highlighting its effectiveness beyond the physiological changes induced by medical interventions.

Earlier investigations have attempted to identify ED patients at high risk of ICU admission by using various variables related to patient characteristics and vital signs. Early warning scores, such as the National Early Warning Score, have demonstrated efficacy in predicting ICU admissions [20]. Machine learning models that incorporate a comprehensive set of variables have also shown strong performance in this task [17]. However, a common limitation among these previously developed models and early warning scores is their reliance on a detailed set of vital sign data for predictions. In cases where patients appear to be relatively stable upon ED presentation and vital sign monitoring occurs at intervals of several hours, risk prediction and the detection of deteriorating patients could be significantly delayed.

Recent technological advances have enabled the acquisition of ECG or PPG signals by using compact wearable devices, such as smartwatches and single-lead ECG devices [15,21]. Our models, which require only beat-to-beat intervals for calculating HR and HRV, are readily adaptable to these devices. This adaptability allows for the monitoring and prediction of ICU admission risk in ED patients even without beds or multiline monitoring devices. A prior study noted that nearly 30% of patients who arrived at the ED with normal vital signs experienced deterioration within 24 h [22]. With respect to overcrowded EDs where continuous bed monitoring may not be feasible, our model can offer a viable solution when used with a wearable device.

### Study Limitations

This study had several limitations that warrant consideration. First, due to the case–control design that was used for developing and validating the models, their prediction probabilities may not be appropriately calibrated. Consequently, recalibration of the models according to the target population for deployment is necessary. Second, compared with patients not under anesthesia, the control group consisted of stable patients who underwent surgery and may have exhibited physiological differences. This approach was adopted in response to the limited vital sign data that are available for stable ED patients, who are typically not subjected to continuous monitoring. Moreover, patients from the OR-VitalDB dataset admitted to the ICU were not included in the case group. This exclusion was due to the routine practice of ICU admission after major surgeries, irrespective of vital signs or health status, and the OR-VitalDB dataset’s lack of detailed reasons for ICU admissions post-surgery. Third, this was a retrospective single-center study; therefore, the results may not be generalizable to different settings. Fourth, we excluded children and elderly patients older than 75 years; thus, the performance of these models in these age groups remains to be validated. Last, our prediction models were limited to incorporating only hypertension and diabetes mellitus as comorbidities, due to the absence of other comorbidity data in the OR-VitalDB dataset. To address some of the aforementioned limitations, the study investigators are currently conducting a prospective validation study in an ED setting.

## 5. Conclusions

In conclusion, the developed models, which incorporate HRV metrics for predicting ICU admission, demonstrated strong predictive performance. The input variables can be easily obtained from a single PPG or ECG sensor, thus offering potential for the models to be used for patient risk monitoring in crowded EDs.

## Figures and Tables

**Table 1:** Demographics and vital sign measurements of patients who were or were not admitted to the intensive care unit.

	Total	Case Group	Control Group	*p*-Value
Number of patients	610	122	488	
Number of data points	10,189	1594	8595	
Age, years	63 (56–69)	63 (56–69)	63 (56–69)	0.78
Sex, male	400 (65.6)	80 (65.6)	320 (65.6)	1.00
Hypertension	198 (32.5)	37 (30.3)	161 (33.0)	0.57
Diabetes mellitus	75 (12.3)	26 (21.3)	49 (10.0)	<0.01
HR, beats/min *	72.1 (64.8–84.4)	105.4 (91.5–116.7)	69.5 (63.3–77.1)	<0.01
SDRR, ms *	61.3 (41.0–86.4)	41.6 (24.3–61.8)	66.2 (45.3–89.2)	<0.01
Adjusted SDRR, ms *	20.6 (−1.5–46.4)	1.8 (−15.5–21.1)	24.4 (3.4–49.2)	<0.01
RMSSD, ms *	59.5 (32.3–89.7)	51.0 (25.6–81.0)	60.3 (35.1–90.2)	0.04
Adjusted RMSSD, ms *	31.2 (6.2–63.0)	24.0 (−1.2–54.4)	33.1 (7.9–65.0)	0.04
Normalized LF power, % *	23.6 (19.0–27.6)	22.4 (16.1–28.8)	23.9 (19.4–27.3)	0.13
Normalized HF power, % *	40.8 (31.9–48.1)	41.3 (31.3–48.1)	40.7 (32.0–48.2)	0.98
LF/HF ratio	0.81 (0.51–1.31)	0.62 (0.42–1.00)	0.87 (0.53–1.39)	<0.01

* The means of the measured HR and HRV metrics for each patient were calculated and compared. Categorical variables are presented as numbers and proportions, while continuous variables are presented as medians and interquartile ranges. Abbreviations: HR, heart rate; SDRR, standard deviation of R-R intervals; RMSSD, root mean square of successive R-R interval differences; LF, low frequency; HF, high frequency.

**Table 2:** Odds ratios for each fixed-effect variable in the GLMM.

	OR (95% CI)
Model 1	
Diabetes mellitus, yes vs. no	3.33 (1.71–6.48)
HR (for every 10 increase)	3.40 (2.97–3.90)
SDRR (for every 10 increase)	0.68 (0.60–0.78)
RMSSD (for every 10 increase)	1.36 (1.22–1.51)
Model 2	
Diabetes mellitus, yes vs. no	3.27 (1.69–6.36)
HR (for every 10 increase)	3.44 (3.00–3.95)
Adjusted SDRR (for every 10 increase)	0.72 (0.63–0.82)
Adjusted RMSSD (for every 10 increase)	1.33 (1.20–1.48)

Abbreviations: OR, odds ratio; CI, confidence interval; HR, heart rate; SDRR, standard deviation of R-R intervals; RMSSD, root mean square of successive R-R interval differences.

**Table 3:** Discriminative performance for each model using data from different time points of the validation set.

	AUC (95% CI)	Sensitivity (95% CI)	Specificity (95% CI)
Model 1			
Randomly sampled data point	0.942 (0.897–0.987)	0.871 (0.753–0.989)	0.910 (0.859–0.961)
First data point	0.921 (0.852–0.989)	0.807 (0.667–0.946)	0.943 (0.901–0.984)
Last data point	0.883 (0.806–0.961)	0.871 (0.753–0.989)	0.812 (0.742–0.881)
Model 2			
Randomly sampled data point	0.947 (0.906–0.987)	0.871 (0.753–0.989)	0.910 (0.859–0.961)
First data point	0.923 (0.855–0.990)	0.807 (0.667–0.946)	0.943 (0.901–0.984)
Last data point	0.886 (0.809–0.962)	0.839 (0.709–0.968)	0.861 (0.799–0.922)

Abbreviations: AUC, area under the receiver operating characteristic curve; CI, confidence interval.

**Table 4:** Discriminative performance for each model using heart rate variability metrics derived from photoplethysmography.

	AUC (95% CI)	Sensitivity (95% CI)	Specificity (95% CI)
Model 1			
Randomly sampled data point	0.928 (0.855–1.000)	0.929 (0.794–1.000)	0.824 (0.755–0.892)
First data point	0.920 (0.828–1.000)	0.929 (0.794–1.000)	0.832 (0.765–0.899)
Last data point	0.911 (0.838–0.984)	0.786 (0.571–1.000)	0.916 (0.866–0.966)
Model 2			
Randomly sampled data point	0.926 (0.853–0.998)	0.929 (0.794–1.000)	0.807 (0.736–0.878)
First data point	0.920 (0.830–1.000)	0.857 (0.674–1.000)	0.899 (0.845–0.953)
Last data point	0.910 (0.835–0.985)	0.786 (0.571–1.000)	0.916 (0.866–0.966)

Abbreviations: AUC, area under the receiver operating characteristic curve; CI, confidence interval.

**Figure A1:**
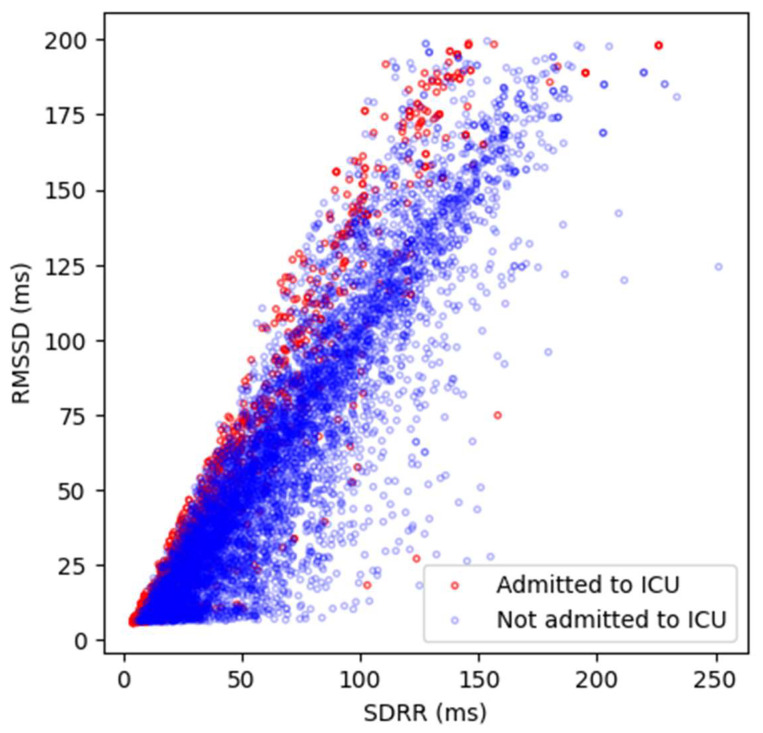
SDRR and RMSSD Values in the Derivation Set According to Intensive Care Unit Admission. Abbreviations: ICU, intensive care unit; RMSSD, root mean square of successive R-R interval differences; SDRR, standard deviation of R-R intervals.

## Data Availability

The data pertaining to the case group in this study are available on reasonable request from the corresponding author. These data are not publicly available due to institutional restrictions on data privacy. The control group in this study used publicly available datasets, which can be found here: https://vitaldb.net/dataset/ (accessed on 20 December 2021).

## Appendix A

Reference Values of SDRR and RMSSD, Adjusting Age and Sex Based on Previous Study

1. Male reference

$${SDRR}_{reference}\left(age\right)=61.5\times {\left(\frac{age}{30}\right)}^{-0.566}(ms)$$

$$RMSSD_{reference}\left(age\right)=44.8\times {\left(\frac{age}{30}\right)}^{-0.804}\left(ms\right)$$

2. Female reference

$${SDRR}_{reference}\left(age\right)=54.7\times {\left(\frac{age}{30}\right)}^{-0.524}(ms)$$

$$RMSSD_{reference}\left(age\right)=43.7\times {\left(\frac{age}{30}\right)}^{-0.666}\left(ms\right)$$

3. Calculation of SDRR and RMSSD values adjusted for age and sex

$${SDRR}_{age\;and\;sex\;adjusted}={SDRR}_{measured}-{SDRR}_{reference}$$

$${RMSSD}_{age\;and\;sex\;adjusted}={RMSSD}_{measured}-{RMSSD}_{reference}$$

## Appendix B

**Table A1:** Demographics and Vital Sign Measurements of Patients in the Derivation and Validation Set.

	Total	Derivation Set	Validation Set	*p*-Value
Number of patients	610	457	153	
Number of data points	10,189	7661	2528	
Age, years	63 (56–69)	63 (56–69)	63 (56–68)	0.44
Sex, male	400 (65.6)	299 (65.4)	101 (66.0)	0.92
Hypertension	198 (32.5)	156 (34.1)	42 (27.5)	0.12
Diabetes mellitus	75 (12.3)	54 (11.8)	21 (13.7)	0.54
HR, beats/min *	74.2 (65.2–87.3)	74.4 (64.9–88.2)	73.0 (66.0–85.4)	0.40
SDRR, ms *	64.2 (40.5–98.7)	63.1 (39.4–97.6)	68.9 (46.8–103.3)	0.16
Adjusted SDRR, ms *	24.4 (−1.5–57.2)	23.6 (−1.9–54.4)	27.6 (2.7–64.5)	0.29
RMSSD, ms *	51.9 (26.5–98.6)	48.9 (26.8–97.6)	59.8 (25.0–103.1)	0.23
Adjusted RMSSD, ms *	23.4 (−1.9–70.4)	20.8 (−1.1–68.8)	31.6 (−2.9–78.3)	0.35
Normalized LF power, % *	22.2 (15.9–29.6)	22.3 (16.3–29.4)	21.7 (14.9–30.4)	0.93
Normalized HF power, % *	40.7 (25.3–51.6)	40.2 (25.1–51.5)	41.9 (25.6–52.5)	0.55
LF/HF ratio	0.62 (0.37–1.30)	0.63 (0.37–1.34)	0.60 (0.37–1.24)	0.68
ICU admission	122 (20.0)	91 (19.9)	31 (20.3)	0.93

* The means of the measured HR and HRV metrics for each patient were calculated and compared. Categorical variables are presented as numbers and proportions while continuous variables are presented as medians and interquartile ranges. Abbreviations: ICU, intensive care unit; HR, heart rate; SDRR, standard deviation of R-R intervals; RMSSD, root mean square of successive R-R interval differences; LF, low frequency; HF, high frequency.

## Appendix D

**Table A2:** Demographics and Vital Sign Measurements of Patients Who Were Intubated and Sedated in the Derivation Set.

	Total	Case Group	Control Group	*p*-Value
Number of patients	375	29	346	
Number of data points	6736	494	6242	
Age, years	63 (56–69)	63 (56–68)	63 (56–69)	0.73
Sex, male	242 (64.5)	21 (72.4)	221 (63.9)	0.36
Hypertension	130 (34.7)	11 (37.9)	119 (34.4)	0.70
Diabetes mellitus	46 (12.3)	7 (24.1)	39 (11.3)	0.04
HR, beats/min *	69.9 (63.8–78.7)	106.5 (93.3–120.2)	69.1 (63.4–76.9)	<0.01
SDRR, ms *	63.7 (42.6–90.3)	41.2 (25.1–53.5)	66.2 (45.0–90.6)	<0.01
Adjusted SDRR, ms *	22.9 (0.9–50.7)	−3.5 (−15.5–16.7)	24.4 (3.8–52.0)	<0.01
RMSSD, ms *	60.2 (33.3–89.8)	44.1 (25.6–67.2)	60.8 (35.1–89.8)	0.08
Adjusted RMSSD, ms *	32.3 (6.7–64.4)	13.2 (−5.7–40.8)	33.3 (8.1–64.5)	0.08
Normalized LF power, % *	23.6 (19.0–26.6)	22.7 (14.2–26.2)	23.6 (19.4–26.6)	0.16
Normalized HF power, % *	40.8 (32.6–47.8)	37.8 (33.5–48.3)	40.8 (32.6–47.8)	0.97
LF/HF ratio	0.82 (0.52–1.28)	0.59 (0.35–0.98)	0.83 (0.54–1.32)	0.02

* The means of the measured HR and HRV metrics for each patient were calculated and compared. Categorical variables are presented as numbers and proportions, while continuous variables are presented as medians and interquartile ranges. Abbreviations: HR, heart rate; SDRR, standard deviation of R-R intervals; RMSSD, root mean square of successive R-R interval differences; LF, low frequency; HF, high frequency.

## Appendix E

**Table A3:** Odds Ratios for Each Fixed Effect Variable in the Sensitivity Analysis.

	OR (95% CI)
Model 1	
Diabetes mellitus, yes vs. no	2.03 (0.27–15.10)
HR (for every 10 increase)	5.35 (3.39–8.44)
SDRR (for every 10 increase)	0.73 (0.51–1.05)
RMSSD (for every 10 increase)	1.19 (0.90–1.59)
Model 2	
Diabetes mellitus, yes vs. no	2.42 (0.34–17.25)
HR (for every 10 increase)	5.23 (3.34–8.19)
Adjusted SDRR (for every 10 increase)	0.75 (0.53–1.06)
Adjusted RMSSD (for every 10 increase)	1.20 (0.91–1.59)

Abbreviations: OR, odds ratio; CI, confidence interval; HR, heart rate; SDRR, standard deviation of R-R intervals; RMSSD, root mean square of successive R-R interval differences.
